# Influence of Food and Beverage Companies on Retailer Marketing Strategies and Consumer Behavior

**DOI:** 10.3390/ijerph17207381

**Published:** 2020-10-10

**Authors:** Amelie A. Hecht, Crystal L. Perez, Michele Polascek, Anne N. Thorndike, Rebecca L. Franckle, Alyssa J. Moran

**Affiliations:** 1 Department of Health Policy and Management, Johns Hopkins Bloomberg School of Public Health, Baltimore, MD 21205, USA; CPerez20@jhu.edu (C.L.P.); AMoran10@jhu.edu (A.J.M.); 2 Westbrook College of Health Professions, University of New England, Portland, ME 04103, USA; mpolacsek@une.edu; 3 Department of Medicine, Massachusetts General Hospital and Harvard Medical School, Boston, MA 02114, USA; athorndike@mgh.harvard.edu; 4 Program in Global Public Health and the Common Good, Department of Biology, Boston College, Chestnut Hill, MA 02467, USA; franckle@bc.edu

**Keywords:** trade promotion, price, promotion, placement, food and beverage, food retailer, grocery, consumer behavior, marketing, chronic disease, choice architecture

## Abstract

The retail food environment plays an important role in shaping dietary habits that contribute to obesity and other chronic diseases. Food and beverage manufacturers use trade promotion—incentives paid to retailers—to influence how products are placed, priced, and promoted in stores. This review aims to: (1) catalogue trade promotion practices that manufacturers use to influence retailer marketing strategies, and (2) describe how these retailer marketing strategies affect consumer purchasing behavior and attitudes. Researchers searched five databases, Academic Search Ultimate, Business Source Ultimate, PsycINFO, PubMed, and Web of Science, to identify literature from industry and academic sources published in English through November 2019. Twenty articles describing manufacturer trade promotion practices were synthesized and provided insight into four types of trade promotion practices: category management, slotting allowances, price discounts, and cooperative advertising. Fifty-four articles describing the impact of retailer marketing on consumers were synthesized and graded for quality of evidence. While comparison across studies is challenging, findings suggest that retailer marketing strategies, such as price promotions and prominent placement, lead to increased sales. Results can guide efforts by policymakers, public health practitioners, and food retailers to design retail environments that improve healthy eating while maintaining retailer financial interests. Additional research should measure the impact of retailer marketing strategies on consumer diet quality and retailer outcomes (e.g., return-on-investment).

## 1. Introduction

The retail food environment plays a critical role in shaping dietary habits and is an important setting for interventions to improve diet quality and prevent diet-related chronic diseases, including diabetes, obesity, and cardiovascular disease [1]. Evidence suggests that marketing of unhealthy foods and beverages may be more common and effective at driving sales compared to marketing of healthy foods and beverages [2,3,4,5,6,7,8,9]. Low-income and racial and ethnic minority populations are disproportionately targeted by unhealthy food marketing, which may exacerbate disparities in diet quality and diet-related chronic disease [10]. For example, advertisements for low-cost, high-calorie, and low-nutrition foods and beverages appear more often in media watched by African Americans [11]; and retailers increase marketing of sugar-sweetened beverages when Supplemental Nutrition Assistance Program (SNAP) benefits are issued each month [12].

Retail food stores, which include both online and brick-and-mortar retailers (see Appendix A for a list of retail formats), are the primary source of food for many populations in both developed and developing economies [13]. In the US, consumers acquire the majority of their calories from supermarkets and superstores [14]. Considering that consumers make an estimated three-quarters of their purchasing decisions while shopping [15], in-store marketing techniques may play an important role in shaping purchase attitudes and decisions [9,16].

Food and beverage manufacturers use trade promotion practices (TPP), or incentives to retailers, to shape in-store marketing [17]. This paper focuses on how TPP influence three out of the “4Ps” of marketing: price, place (both the channels through which products are sold and where products are placed in stores), and promotion (efforts to engage consumers and communicate product features, such as signs) [18]. The fourth “P” of marketing, “product,” is less frequently shaped by TPP, but rather by manufacturers in-house, through efforts such as packaging and product formulation. Similarly, TPP more commonly shapes where items are placed in stores and on shelves (i.e., product placement) rather than the channels through which products are sold. Food and beverage manufacturers allocate about $1 trillion annually to TPP—between 50 and 70% of their marketing budgets and nearly 20% of their total revenue [17,19].

There is growing interest among policymakers, researchers, advocates, and retailers in creating policies and corporate practices that promote healthy food retail. To inform efforts to improve the food retail environment, it is important to understand (1) the types of TPP currently used by food and beverage manufacturers to influence retailer marketing strategies, and (2) how retailer marketing strategies, in turn, affect consumers. The first part of this research question—which types of TPP are used to influence retailers—is understudied, particularly in the public health literature. A 2016 investigative report commissioned by the Center for Science in the Public Interest, which describes TPP but did not use a systematic approach to gather data or survey the literature, served as a launching point for this aim [17].

The second part of this research question—how retailer marketing strategies impact consumers—has been only partially explored in previous reviews. Specifically, three previous reviews have focused on price promotions’ impact on consumers; all three concluded that price promotions were associated with consumer behavior [3,9,20]. In a 2012 integrative review, Glanz et al. synthesized literature on the impact of price, placement, and promotion on consumer behavior but limited their search to literature focused on brick-and-mortar grocery stores. They found that all three marketing strategies were associated with increased product liking and purchasing, with some variation in degree of impact by strategy [21]. This review serves as an update to and expansion of the Glanz et al. review, synthesizing literature since 2011 and including other nontraditional retail settings such as online retailers and convenience stores. This review focuses on identifying, where possible, whether and how outcomes differ when healthy versus unhealthy products are marketed. Findings from this study can inform efforts by advocates, policymakers, public health practitioners, and food retailers to design food retail environments that promote healthy eating while maintaining retailer financial interests. This study will also identify gaps in the literature and provide directions for future research.

## 2. Methods

Two research questions were identified: (1) how do food and beverage manufacturers use TPP to influence retailer marketing strategies; and (2) how do retailer marketing strategies impact consumer purchasing behavior and attitudes? Searches were conducted for peer-reviewed and grey literature (e.g., conference abstracts and proceedings, reports, dissertations) in English. To identify publications from diverse disciplines including public health, business, economics, marketing, and social sciences, the following databases were searched: Academic Search Ultimate, Business Source Ultimate, PsycINFO, PubMed, and Web of Science. Search terms for each research question were developed by the study authors in consultation with industry and academic experts and a research librarian (Appendix B). The selection and analysis of the results were carried out under the Preferred Reporting Items for Systematic Reviews and Meta-Analyses (PRISMA) guidelines [22].

### 2.1. Research Question 1: Search Strategy and Inclusion Criteria

**Figure 1:**
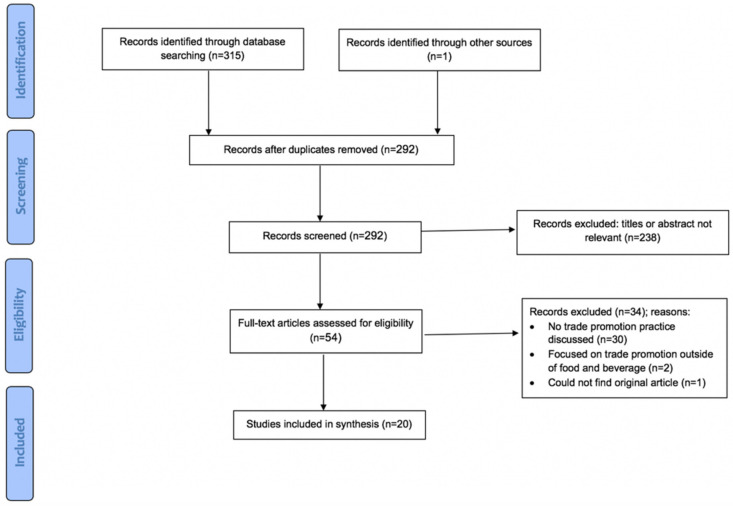
Preferred Reporting Items for Systematic Reviews and Meta-Analyses (PRISMA) Diagram for Research Question 1.

To answer the first research question, a narrative review was conducted to identify and catalogue types of trade promotion practices used by food and beverage manufacturers to influence retailer marketing strategies (Figure 1). Articles published through November 2019 were included. Article titles and abstracts were independently screened by two authors (AH and CP) for inclusion. Full-text review was completed by the first author (AH). Any questions about study inclusion were resolved through discussion with the second author (CP).

### 2.2. Research Question 2: Search Strategy and Inclusion Criteria

**Figure 2:**
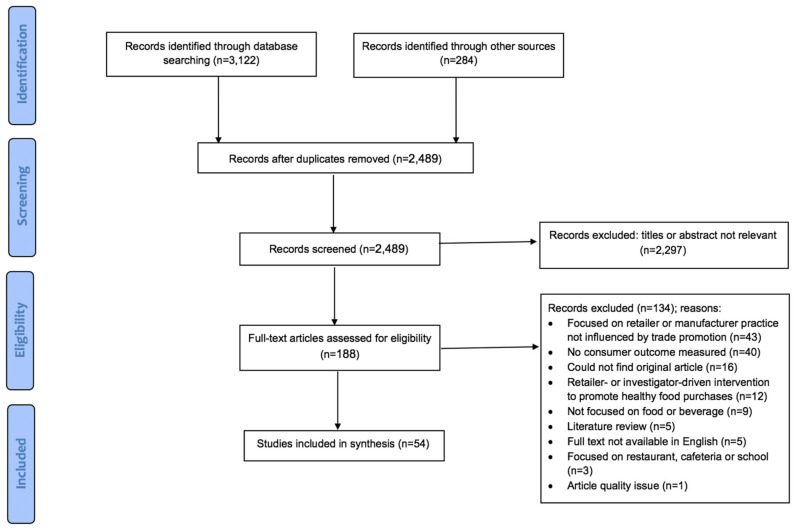
PRISMA Diagram for Research Question 2.

To answer the second research question, a systematic review was conducted to understand the impact of retailer marketing strategies on consumer behavior and attitudes (Figure 2). Inclusion criteria were that the article must (1) be published between January 2011 and November 2019 (to capture studies published since the Glanz et al. review) and (2) measure the impact of retailer marketing strategies influenced by TPP on consumer purchasing behavior or attitudes. Studies were excluded if they assessed (1) an investigator-driven healthy retail intervention (review by Karpyn et al., forthcoming); (2) retailer or manufacturer practices unrelated to TPP (e.g., product labeling); (3) restaurants, vending machines, cafeterias, or schools, or (4) were not original research (e.g., literature reviews). The excluded literature reviews were incorporated into the background and discussion section of this review. Two authors (AH and CP) independently reviewed titles, abstracts, and full texts for inclusion and met to reconcile differences. Reference lists of included articles were also scanned, and relevant articles included.

### 2.3. Quality of Studies

The quality of included studies for the second research question was assessed using the Newcastle–Ottawa quality assessment scale, adapted for cross-sectional studies [23] (Appendix C). The Newcastle–Ottawa scale assigns studies composite quality scores by awarding a certain number of stars out of a total of nine possible stars. Similar to an approach used by Bennett et al., amendments were made to the scale; for articles using aggregate sales data, a “not applicable” option was allowed for categories of “non-respondents” and “controlling for confounding variables” [3]. The denominator (total number of possible stars) was reduced appropriately. Two authors (AH and CP) independently graded the included studies and met to reconcile differences. As described by Takehashi and Hashizume [24], studies that earned fewer than a third of the possible stars were classified as low-quality studies.

## 3. Results

### 3.1. Narrative Review of Trade Promotion Practices

Twenty articles were identified that described TPP used by manufacturers to influence retailer marketing strategies [25,26,27,28,29,30,31,32,33,34,35,36,37,38,39,40,41,42,43,44]. Of these, 13 articles were published in the peer-reviewed literature or through conference proceedings and seven articles were published in trade publications. Of peer-reviewed publications, two were in public health or public policy journals and the remainder were in journals focused on retail, economics, or marketing. Thirteen articles focused on the US, six focused on other countries, including Brazil, the United Kingdom (UK), New Zealand, Sweden, Finland, Italy, and Portugal, and one used a global perspective.

Results indicate that manufacturers use four types of TPP to shape retailer marketing strategies: (1) category management, (2) slotting allowances, (3) price discounts, and (4) cooperative advertising (Table 1). These terms may differ across retailers, manufacturers, and countries; for example, in Europe, slotting allowances are also referred to as listing charges [25]. Certain types of TPP may be used more often for some product categories and in some retail formats. For example, slotting allowances are more often used in highly concentrated, processed product categories such as beverages, snacks, and candy [32]. In smaller stores, such as convenience and corner stores, more informal incentive-based agreements between suppliers and retailers are common [36].

#### 3.1.1. Category Management

Eleven articles focused on category management [27,28,31,34,35,37,38,39,41,43,44]. Category management is the collaboration between manufacturers and retailers to make decisions regarding product assortment, space allocation, pricing, and in-store promotion for entire product categories. Categories (e.g., ice cream, yogurt) are treated as strategic business units to ensure maximum efficiency and boost sales for the whole category, rather than for individual brands [27]. Category management typically uses a shopper-centric and research-based approach to promote consumer satisfaction and loyalty [39,41,44].

A leading manufacturer in a category often serves as the “category captain,” overseeing category management and customizing plans on a store-by-store basis. Such an arrangement is often considered beneficial for both retailers and manufacturers: it allows retailers to concentrate on other aspects of their business, and manufacturers to focus on increasing category market share and profitability [28]. While some retailers have safeguards in place to ensure category captains are not unfairly advantaged, critics contend that because category captains have influence over which brands and products within a category are stocked and promoted, category captains may be able to exclude competitors [28,43].

#### 3.1.2. Slotting Allowances

Seven articles focused on slotting allowances, or lump-sum fees paid by manufacturers to retailers in exchange for access to the consumer market (i.e., shelf space) [25,29,32,33,36,40,42]. These include slotting fees to introduce a new product onto shelves, pay-to-stay fees to maintain shelf position, floor fees to make sales presentations and offer in-store samples, and display fees, which may cover premium placement, display materials (e.g., wire racks, prefabricated displays), and promotional signage. Theoretical explanations for why slotting allowances have become widely used include a market power explanation (i.e., slotting allowances reflect growing power among retailers who control access to the market) and an efficient market explanation (i.e., slotting allowances enable efficient allocation of scarce shelf space) [25]. According to the efficient market rationale, slotting allowances help retailers defray the costs and risks associated with new product introductions in light of an estimated 70% failure rate for new products [45]. Evidence suggests that slotting allowances in the US alone total between $6 billion and $18 billion per year [25,46]. Nationwide introduction of a new product in the US can cost up to $1–2 million in slotting fees [45]. In countries with more independent retailers, slotting allowances are less common.

#### 3.1.3. Price Discounts

Four articles discussed price discounts that manufacturers provide to retailers to incentivize retailers to stock, display, or provide promotional discounts for their products [26,33,36,47]. Manufacturer discounts may be fixed or performance-based [47]. Fixed discounts are price reductions offered to the retailer on a per unit or per case basis, often at the time of billing, for a limited period of time. Performance-based discounts are tied to a measure of retailer performance such as number of units sold, displayed, or offered on price promotion. Discounts may be passed on to the consumer in the form of temporary price reductions (TPR) or coupons, affecting final sale prices [33,47]. Manufacturers may also provide retailers products for free to encourage retailers to stock new products or provide customer discounts, giveaways, or in-store samples [26,36].

#### 3.1.4. Cooperative Advertising

One article focused on cooperative advertising. Cooperative advertising is the collaboration between manufacturers and retailers to create and distribute local promotional materials such as newspaper inserts or direct mail flyers [42]. A cooperative advertising agreement may be initiated by either a retailer or manufacturer. Typically, the manufacturer will design the promotional materials, providing product images and templates, and the manufacturer and retailer will share the cost of printing and distribution.

### 3.2. Literature Review of Impacts of Retailer Marketing Strategies on Consumers

Fifty-four articles that describe the impact of retailer marketing strategies on consumer behavior or attitudes were identified (Table 2). These included peer-reviewed literature (*n* = 44), dissertations (*n* = 4), conference proceedings (*n* = 3), reports from government or industry (*n* = 2), and trade publications (*n* = 1). Studies occurred in the US (*n* = 17), UK (*n* = 11), other European countries (*n* = 8), Asian and Middle Eastern countries (*n* = 8), Australia or New Zealand (*n* = 6), Canada (*n* = 1), and Egypt (*n* = 1); two articles did not specify location. Articles focused on a range of retail formats, including supermarket/grocery stores (*n* = 43); convenience/corner stores (*n* = 9); online retailers (*n* = 4); dollar stores (*n* = 1); other (e.g., organic markets, liquor stores, pharmacies, *n* = 9); and four articles did not specify the retail format assessed. Ten articles evaluated multiple retail formats. Data sources used varied widely; scanner or panel data was the most commonly used data source (e.g., Kantar Worldpanel data) (*n* = 26), followed by customer survey (*n* = 21), direct observation (*n* = 9), customer interviews or focus groups (*n* = 8), marketing data from the manufacturer or retailer (*n* = 5), retailer loyalty card data (*n* = 4), and other data sources (e.g., customer diaries, eye scanner, store audits, bag checks, *n* = 6); one article did not specify the data source used. Nearly one third (*n* = 17) used multiple data sources. No articles declared conflicts of interest.

TPP influence three categories of retailer marketing strategies: how products are priced, placed, and promoted. Results below are organized according to these three domains. Notably, comparison across studies is challenging given they focus on different products, use different study designs, and employ different outcome measures. The two final sections of the results describe findings from studies that compare outcomes across two or more retailer marketing strategies and compare the impact of marketing of healthy versus unhealthy products.

#### 3.2.1. Pricing

Retailers employ a variety of price promotion strategies, including coupons, bundle deals (e.g., buy-one-get-one, 2-for-1), and TPR (also called rollbacks). In the US and the UK, an estimated 40% and 34% of all purchases are price promoted, respectively [6,48]. Estimates indicate that between 24% [49] and 67% [4] of unhealthy foods and beverages are purchased while price promoted, though prevalence of promotions differ across retailer formats and neighborhood [4]. A review of price promotions among Scottish retailers found that TPR are the most prevalent form of price promotion, accounting for 74% of promotions, followed by bundle deals, which represent 23% of promotions [9]. Price promotions are offered more frequently for unhealthy compared to healthy products [2,3,5,7,8,9,20,50,51].

Thirty-two articles focused on price promotions. Eight presented results separately for coupons, seven presented results separately for TPR, and the remainder did not specify the type of price promotion assessed or assessed multiple types of price promotion and did not present results separately.

##### Coupons

Coupons may be distributed by retailers or manufacturers. In 2017, 302 billion coupons for consumer packaged goods were distributed in the US [52]. Six studies evaluated coupons and reported coupons were associated with increases in overall purchase volume, impulse purchase volume, brand choice, and product trialing (first-time purchase), but not brand loyalty [53,54,55,56,57,58]. Two studies assessed customized coupons, which target consumer groups based on demographic characteristics or past shopping behavior, and found they were associated with increased purchasing of targeted products [54,56]. Coupons in some product categories may be more impactful than others: one study found that coupons led to greater product trialing when promoting leading brands and categories that were popular, easy to store, had fewer products in the category (easier for customers to process less options), and were frequently on sale [55]. Another study found that while customized coupons led to increased purchases for both healthy and unhealthy products, they were more effective for unhealthy products [54].

##### Temporary Price Reductions

All eight studies that evaluated TPR detected associations with one or more consumer shopping behaviors, including purchase volume, impulse purchase volume, brand choice, and brand market share [58,59,60,61,62,63,64,65]. TPR may have a stronger impact on some outcomes compared to others: one study that assessed wine purchases in the UK found that TPR strongly influenced brand selection, somewhat influenced purchase volume, but did not influence purchase initiation [61].

Three articles assessed the impact of TPR in online retail [63,64,65]. Two out of three studies found that online price promotions were associated with increased purchases [63,64]; the third found no association [65]. One of the two studies that detected an association reported that because online purchases were delivered, barriers to stockpiling were eliminated, resulting in increased purchase volume compared to in traditional brick-and-mortar retail outlets [64]. The other reported that when a retailer with both online and brick-and-mortar retail outlets offered price promotions online, online sales increased, but sales in the brick-and-mortar stores decreased [63]. That study also found that high frequency of online promotions led to diminished effects over time [63].

##### Other Price Promotions

Thirteen articles on price promotions did not specify the type of price promotion studied or examined several types of price promotions together [6,8,50,66,67,68,69,70,71,72,73,74,75]. Many studies using panel data were unable to distinguish between types of price promotion used by customers. All studies identified positive associations between price promotions and one or more outcomes, including purchase volume, stockpiling purchase volume, purchase initiation, product trialing, and store choice. Within some studies, however, price promotions were positively associated with some outcomes and not others. For example, one study assessing Japanese market trends over time found that manufacturer expenditure on sales promotion was associated with an increase in total purchase volume but a decrease in manufacturer profits [66]. Another study found that price promotions led to short-term sales increases, but in more than half of cases, did not increase category revenue due to brand-switching (substitution) effects within the category [70].

Quantitative estimates on the impact of price promotions are difficult to compare because researchers used different outcome measures. Three studies, all using data from the Kantar Worldpanel, illustrate this challenge [6,8,69]. Nakamura et al. estimated that a 1% increase in price discount led to a sales uplift of 1.44% within a given category [6]. Smithson et al. found that approximately one-fifth of foods and beverages bought on price promotion were purchased in addition to what would be expected absent a price promotion, leading to an overall increase in food and drink purchase volume [8]. Revoredo-Giha et al. found that the presence of a price promotion increased spending between 2% and 10%, depending on the product category [69].

The effect of price promotions may differ across product categories and consumer characteristics. For example, one study found that while, price promotions did not, on average, affect beef sales, they did influence sales for certain cuts of meat and consumer groups (e.g., young families versus older adults) [68]. Another study found that price promotions were associated with increased soda sales across all levels of consumer education and retail formats, but the effect was weaker in neighborhoods with a higher proportion of residents with at least a post-secondary certificate or diploma [71].

Three studies compared differences in the impact of price promotion on healthy and unhealthy products [67,69,74]. Two of these studies found that purchase volume increased as price decreased for unhealthy foods but not for healthy foods [67,74]. Another, however, found that price promotions led to increases in total spending and spending by category for both healthy and unhealthy foods, though the effect was greater for less healthy foods [69]. Specifically, they found greater increases in spending for unhealthy categories such as confectionery (10%) and beverages (9%) and smaller increases for healthier categories such as fruits and vegetables (5%), grains (3%), and dairy (2%).

##### Perceived Importance of Price Promotions

Eight articles assessed consumer perceptions regarding the importance of price promotions in shaping their purchasing decisions [67,73,76,77,78,79,80,81,82]. Though the populations and contexts assessed varied across articles, all studies found that shoppers considered price promotions to be an important factor influencing their shopping behavior. Three of these studies assessed perceived importance of price promotions within specific cultural and religious contexts. In one study, Egyptian Muslim shoppers reported that TPR and bundled deals led them to engage in more stockpiling and spending, but other discount promotions considered not compliant with Shari’ah law, such as sweepstake draws and scratch-and-win promotions, did not shape their behavior [79]. In a study of Pakistani Muslim shoppers, participants reported that their intentions to purchase Halal products were shaped by price promotions [80]. Through interviews with “ethnic” shoppers in the UK, a final study found that participants reported diverse responses to price promotions, ranging from responsive to hostile, depending on the perceived “net worth” of the promotion [81].

#### 3.2.2. Placement

Sixteen studies focused on how products were placed within stores, measuring visual attention, purchase volume, or spending as the primary outcomes [15,46,58,62,83,84,85,86,87,88,89,90,91,92,93,94]. Through slotting allowances and category management, manufacturers are able to secure placement in premium store locations, including on the endcap (i.e., end-of-aisle displays free from direct aisle-based competition), in the checkout aisle, and on freestanding displays. In 2012, an estimated 60% of products in stores were cross-promoted, meaning they are were displayed in secondary locations away from their “home” aisle [15]. Displays may be located anywhere in the store: approximately 42% of displays are located on the endcap, 28% in the aisle, 23% on the perimeter of the store, and 7% at the front of the store [15]. In an evaluation measuring shoppers’ visual attention, 13% of all eye-fixations were drawn to in-store displays; of these, 44% were to endcaps, 34% to floor stands, 12% to in-line displays (i.e., gondola, or freestanding wire or metal shelving), and 10% to power wings (i.e., sidekick displays, or cardboard displays that attach to shelving) [15].

##### Endcaps

Five studies focused on placement in endcaps; all found significant positive effects on purchasing [46,83,85,86,87]. In a study of UK stores, endcap displays led to increased purchase volume for beer by 23.2%, for wine by 33.6%, for spirits by 46.1%, and for carbonated drinks by 51.7%; sales uplift was even greater for tea and coffee [85]. Two studies found that endcaps located at the rear of the store are more impactful than those at the front of the store [47,91]. In an experimental study in Australian grocery stores, placement of unhealthy products on rear endcap displays generated a 416% uplift in sales, while placement on front endcap displays generated a 346% uplift in sales [46]. Findings also suggest that endcaps are most impactful when located away from in-store sampling [87] and in stores without middle, perpendicular aisles [86].

##### Shelf Placement and Space

Category management and display fees can also affect where categories are placed within a store, and where individual products are placed on shelves (e.g., at eye-level for adult shoppers). Three experimental studies suggest that placement at the front of the store, in central aisles, at eye-level, and away from other popular categories can have positive effects on sales [88,91,92]. In one study, moving fruits and vegetables to a prominent location at the front of a grocery store led to an increase in sales volume and spending on fruits and vegetables [92]. In another, placement of dairy products in a central aisle was associated with increased product sales and purchase incidence, while placement next to popular categories had an “attention stealing” affect, leading to decreased sales [88]. In a laboratory-based study of college students, junk food items placed at eye-level received more visual attention than those on higher or lower shelves [91]. However, an observational study in New York City bodegas found no association between unhealthy beverage purchases and the placement of healthy products in prominent locations (i.e., water at eye-level and produce in at the front of the store) [89].

Total amount of dedicated shelf and display space (measured in feet) was associated with increased sales in two studies [84,90]. In one study, Minneapolis stores with more shelf space dedicated to fruits and vegetables had healthier purchases (i.e., more fruits and vegetables, more whole grains, and higher healthy eating index scores) [84]. Similarly, in a study of Hispanic shoppers in San Diego tiendas, each additional square foot of display space for fruits and vegetables was associated with a $0.02 increase in weekly amount spent on fruits and vegetables [90].

##### Other Placement Strategies

Four additional studies evaluated the impact of placement but did not specify how or where evaluated products were displayed [58,62,93,94]. All four studies found that presence of displays was positively associated with impulse purchase volume, spending, or brand choice. One of these studies was an industry report that assessed a multifaceted marketing campaign, however, and it is unclear what proportion of the sales uplift was attributed to placement [93].

#### 3.2.3. Promotion

Sixteen articles focused on promotion [53,73,76,80,82,84,87,89,90,95,96,97,98,99,100,101]. Manufacturers use cooperative advertising and display fees to secure promotional signage, in-store sampling (i.e., taste tests), loudspeaker announcements, games, and other giveaways.

##### Signs

All three studies that measured the relationship between signs on shelf facings (called shelf-talkers or aisle violators) and purchase behavior focused on promoting healthy products; none detected a significant association [84,89,90]. In tiendas in San Diego, the number of signs promoting fruits and vegetables was not associated with fruit and vegetable purchases among Hispanic consumers [90]. In Minnesota stores, healthy advertising inside stores was not associated with purchasing, and, in fact, healthy advertising outside stores was associated with less healthy purchases [84]. In New York City bodegas, neither signs advertising water nor signs advertising sugar-sweetened beverages were associated with sugar-sweetened beverage purchases [89].

One study assessed “feature advertising“ in two competing grocery stores, but did not describe components of “feature advertising” [73]. This study found that feature advertising led customers to choose to shop at the store with featured advertising over another store.

##### In-Store Sampling

In-store sampling was found to be associated with greater brand loyalty and purchase volume in three studies [53,87,99]. Several factors may moderate the impact of in-store sampling on purchases: studies suggest that benefits are maximized when the product being offered on sample matches the product displayed on the closest endcap [87,99]. One study also found a sales increase when in-store samples were offered close to the weekend compared to earlier in the week, when store personnel were present to offer the sample (24.3% increase compared to without store personnel present), when there was a sign promoting the product (90.8% increase compared to no sign), and when a commercial for the product is played on an in-store TV (36.3% increase compared to no commercial) [99].

##### Games, Giveaways and Limited-Time Offers

Findings on the impact of games, giveaways, and limited-time offers differed across studies [53,82,100]. In one study, customers reported that in-store games and lotteries led to greater customer loyalty and stronger relationships with promoted brands [53]. In another study, giveaways of collectible items increased the probability of brand choice and category purchase incidence, particularly when paired with a price discount, but did not change the purchase volume decision [100]. In a final study, both limited-time and membership deals were found to increase purchase incidence in an organic market [82].

##### Perceived Importance of Promotions

Seven articles assessed consumer perceptions regarding the importance of promotional activities in shaping their purchasing decisions [76,80,95,96,97,98,101]. Studies investigated different types of promotions and used different methods to assess customer perceptions, and found varying levels of perceived importance. Five studies found that consumers reported high levels of perceived importance of marketing on their attitudes toward purchasing [80,96,97,98,101]. Two studies, however, found promotional offers to be less persuasive: in a survey of Australian shoppers, 41% said they were influenced by promotional offers, but, in focus groups and interviews, many said that while promotional offers engaged them initially, trust and emotional connection to the brand was the primary driver of their purchase decisions [95]. In a survey of Vietnamese urban shoppers, participants described merchandise display and promotion as the least important factor from a list of seven factors influencing impulse purchase behavior [76].

#### 3.2.4. Comparison of Marketing Healthy versus Unhealthy Products

As previously described, a small number of studies compared marketing of healthy versus unhealthy products [54,67,69,74,84,89,90]. Of these, four focused on price, three on placement, and three on promotion. Half of price-focused studies found that price promotions led to increased purchasing of unhealthy but not healthy products, [67,74] whereas the other half of studies found that while the effect was stronger for unhealthy products, price promotions led to increased purchasing of both healthy and unhealthy products. One of the three studies focused on placement found no association between prominent placement of healthy products and purchasing [89]. The other two studies, however, found that stores with more shelf and display space dedicated to fruits and vegetables had healthier sales [84,90]. Notably, both of these studies were cross-sectional and thus were unable to determine causality. Finally, none of the three studies focused on signs promoting healthy products detected a relationship with purchasing [84,89,90].

#### 3.2.5. Comparison across Marketing Strategies

A small number of articles directly compared one retailer marketing strategy to another. Four of these asked participants to rank factors that shape their purchasing; in all four, participants reported that price promotions were the most or one of the most influential factors shaping their attitudes toward purchasing. Vietnamese shoppers reported that price promotions influenced their spontaneous purchase tendencies more than displays [76]. Taiwanese organic market shoppers reported that discounts and free giveaways impacted their shopping behavior more than membership or limited-time offers [82]. Two other studies focused on Muslim shoppers: in one [80], shoppers reported being equally influenced by Halal marketing promotions and pricing, while in the other [79], shoppers reported price discounts influenced their purchase intention more than giveaways, games, and in-store samples.

One study compared different types of price promotions, finding that sensitivity to coupons was greater than sensitivity to TPR [58]. The remaining studies quantitatively compared price promotions to either promotion or placement; results largely indicated that price promotions are more impactful than other types of marketing strategies [53,59,73,85]. Specifically, one study found that price was a stronger driver of stockpiling purchases than feature and display promotion [73]. Another found that a 20% TPR increased fair trade coffee sales more than providing information or a moral appeal [59]. Another study found that the effect size for endcap placement was equivalent to a price decrease for alcohol categories of between 4% and 9% per volume, and a price decrease for non-alcohol categories of between 22% and 62% per volume [85]. One study, however, found that price promotion and in-store sampling produced different benefits: in-store sampling helped nurture consumer loyalty more than coupons, but coupons resulted in more purchases [53].

#### 3.2.6. Quality of Evidence Grading

On average, included studies received 65% of total possible stars (Appendix D). Only three of the 54 studies included in this review were of low-quality, having earned less than a third of all possible stars. The two categories in which studies most often earned zero stars were sample size (*n* = 27) and non-respondents (*n* = 28). Nearly half of the included articles omitted sample size calculations or justification; this was particularly common among studies using questionnaires or published in non-peer-reviewed sources. Only one study compared respondents and non-respondents or reported their response rate, though for 25 articles, this information was considered not applicable due to use of panel data. More than half of all studies earned the maximum number of stars in the assessment of outcome category by linking records or using an independent blind assessment to determine the outcome.

## 4. Discussion

This review is the first to synthesize literature from academic and industry sources on the approaches that manufacturers use to shape retailer marketing strategies, and, in turn, consumer behavior and attitudes. More than half of the included studies focused on pricing; fewer articles assessed placement or promotion and many of these articles focused on purchase attitudes rather than behavior.

Findings suggest that all types of price promotions, including coupons, multi-buys, and TPR, shape purchasing behavior. Placement in premium store locations, such as on endcaps, and in-store samples are also effective drivers of sales. Other promotion activities, such as giveaways, games, and signs, may be less impactful. Notably, findings suggest that retailer marketing strategies may be less effective at driving sales for healthy foods and beverages [54,67,69,74,84,89,90]. Of the small number of studies that specifically considered sales of healthy products, the majority found that retailer marketing strategies, including signs and price promotions, were not associated with increased sales of healthy products. Two studies did find increases in healthy purchases, but effect sizes were smaller than for unhealthy products [54,69]. Previous reviews have similarly found that promotions of unhealthy products are more impactful than those for healthy products [4,9]. Studies of retailer- or investigator-driven interventions to specifically promote healthy purchases, however, were outside the scope of this study (these interventions are reviewed by Karpyn et al. in a paper published as part of this special issue) and may have identified retailer marketing strategies that effectively increase healthy purchases.

Findings regarding the impact of price promotions and product placement on consumer behavior are consistent with findings of previous reviews [3,9,20,21]. To the authors’ knowledge, the review by Glanz et al. is the only study to also explore promotion; however, they did not identify any studies related to signage, and only one related to in-store sampling [21]. Several previous reviews, including Glanz et al., excluded studies in nontraditional retail settings, such as convenience and dollar stores and online retail. Findings from included studies of nontraditional retail formats suggest that retailer marketing strategies have similar effects across retail settings. One notable difference, however, is that consumers may be more likely take advantage of promotions in online retail by stockpiling, as they are not required to transport their purchases home themselves [64].

While only three studies were rated as “low quality,” analytic rigor and rigor of data sources in included articles varied widely. Many articles from industry publications did not describe their analytic methods; thus, it was challenging to assess the quality of evidence of these articles. Additionally, while no studies listed conflicts of interest and many did not disclose funding sources, several were written by industry representatives and published in trade publications and may, therefore, have been more inclined to include findings that portrayed the companies favorably. A growing number of studies used store scanner and loyalty card data; these data sources, which provide large sample sizes and detailed sales information, should be used widely, particularly when paired with information on customer demographics. Several of the included studies occurred in controlled, laboratory settings; strategies that proved impactful in these settings, such as placement of products at eye-level, should be adapted and tested in real-world retail environments.

Study findings point to strategies that policymakers, public health practitioners, and retailers can use to ensure that retail environments promote healthy eating. Results suggest that policies and corporate practices that limit promotion of unhealthy products, rather than interventions to promote healthy products, may be needed to improve diet quality. Policies and practices can target each of the four TPPs identified in this study to curb promotion of unhealthy products. For example, policies could prohibit category captains from excluding competitors, or create healthy checkout aisles by prohibiting retailers from accepting stocking fees to display ultra-processed foods in checkout aisles. Considering SNAP is an important revenue stream for many US retailers, restrictions on promotion of unhealthy products could also be integrated into requirements for SNAP-authorized retailers [102].

### 4.1. Future Research Directions

Findings from this study highlight directions for future study. Research is needed to evaluate:

1. **Online and other nontraditional retail formats.** Eighty percent of included articles focused on retailer marketing strategies in grocery stores and supermarkets; other nontraditional retail formats such as dollar stores and online retailers should be assessed. Despite rapid proliferation of dollar stores in the past decade [103], they were assessed in only one of the included articles. Considering dollar stores are most common in rural and low-income communities, evaluations in dollar stores may provide insight into geographic and socioeconomic disparities in diet and food purchasing. In 2015, dollar stores represented two-thirds of new stores in designated “food deserts” [104]. Relatedly, online retail was the focus of only four of the included articles. While online grocery retail represented only 6.3% of total US grocery spending in 2019, [105] online sales are rapidly expanding, and due to concerns about COVID-19 transmission, are expected to grow more than 40% in 2020 [106].
2. **Distal consumer outcomes including consumption and health.** None of the included studies measured the impact of retailer marketing strategies on distal or long-term outcomes, such as diet quality or weight. Admittedly, it may be difficult to detect the impact of marketing strategies on health outcomes, especially because diet-related health outcomes are influenced by a multitude of environmental and biological factors. Dietary consumption, which has been linked to health outcomes in the public health literature, however, may be assessed. Analysis of these outcomes will require collection of different types of data, such as food frequency questionnaires or dietary recall surveys, coupled with objective purchase data. Dietary data collection methods, however, do have limitations (e.g., food frequency questionnaires may not be sensitive enough to detect small effect sizes, and dietary recalls are resource-intensive and subject to recall bias).
3. **Other outcomes of importance to retailers and manufacturers.** While this review excluded studies that did not measure consumer behavior or attitudes, the initial scan of titles and abstracts revealed few studies that assessed other outcomes of importance to industry, such as short- and long-term return on investment and customer lifetime value (i.e., the total profit a retailer makes from customers over their lifetime). Interventions that benefit public health, in order to be sustainable and acceptable to manufactures and retailers, must consider these outcomes.
4. **Differential impacts of retail practices on consumers by demographic characteristics.** Few studies compared how retailer marketing strategies affected different consumer segments, such as families with children, shoppers with low income, or shoppers who identify as racial or ethnic minorities. Insight into how certain populations may be disproportionately influenced or targeted by retailer marketing strategies can guide intervention efforts.
5. **Retailer marketing strategies that have the strongest impact on consumer behavior.** Only a small number of studies directly compared the impacts of different retailer marketing strategies, and most of these focused on perceived importance. Additional head-to-head comparison of retailer marketing strategies is needed to prioritize which components to include in future interventions.
6. **Trade promotion practices that have the strongest impact on retailer behavior.** Data on TPP are largely proprietary, and thus, research is limited on the amount manufacturers spend annually on TPP, which TPP are used most frequently, what proportion of retailer profit comes from TPP, and which TPP are the strongest drivers of retailer marketing. Additional research, potentially done in partnership with industry, is needed to understand these powerful market drivers.

### 4.2. Limitations

It is possible that different search terms or databases might have identified further studies. The quality of evidence grading tool was adapted from the Newcastle–Ottawa quality assessment scale, which was initially designed for case-control and cohort studies. Thus, it was challenging to assess quality of evidence for qualitative and observational studies, as well as industry reports and news articles, which provide few details on methods. Additionally, while study inclusion criteria were designed to capture studies from any country, only studies published in English were included. The majority of identified studies focused on the US and UK, and many countries were not represented in these findings. As a result, results may not be generalizable across countries and cultures.

## 5. Conclusions

This review finds evidence that by influencing retailer marketing strategies through TPP, manufacturers can shape consumer behavior and, ultimately, diets. The 74 studies included in this review suggest that TPP have a considerable effect on product placement, pricing, and promotion, and, in turn, on a range of customer outcomes, including purchase volume, spending, and attitudes. Findings point to a particularly strong relationship between price promotions and consumer behavior and differential impacts by product type and consumer characteristics. This review builds on previous work by synthesizing findings from recent studies and studies focused on non-traditional retail formats. Study findings provides valuable insight that can guide efforts by policymakers, public health practitioners, and food retailers to design retail environments that promote healthy eating. Public health practitioners and policymakers could consider policies that regulate promotion of unhealthy products by targeting each of the four TPPs identified in this study. Further investigation is warranted to determine the impact of retailer marketing on dietary outcomes and outcomes of importance to retailers. Further research is also needed in online and nontraditional retail settings.

## Figures and Tables

**Table 1:** Definitions of trade promotion practices.

Trade Promotion Practice (*n*)	Definition
Category management (11)	Collaboration between retailers and manufacturers to make decisions regarding product assortment, supply, pricing, and promotion for entire categories
Slotting allowances (7)	Lump-sum fees paid by manufacturers to retailers in exchange for access to the consumer market (e.g., shelf space, prominent placement)
Price discounts (4)	Fixed discounts (merchandise is sold at a set discount for a specified period) or performance-based discounts (discounts are tied to a measure of performance such as units sold or displayed)
Cooperative advertising (1)	Cost-sharing between retailers and manufacturers to create and distribute promotional materials

Note: some articles discussed multiple trade promotion practices, so ns sum to greater than the total number of included articles.

**Table 2:** Study design, marketing strategy, retailer format, country, study duration, data source, objectives, outcomes, and key findings for studies included in research Question 2 (*n* = 54).

Reference	Marketing Strategy	Retail Format	Country	Study Duration	Data Source	Objective	Outcome	Key Findings
Andorfer, et al. (2015) [59]	Price	Supermarket/ grocery store	Germany	5 mo (5 March, 2012– 29 July 2012)	Scanner/panel data Customer surveys Direct observation	To identify how information, price, and moral considerations influence consumers’ purchases of fair trade (FT) coffee products.	Purchase volume Purchase frequency	- A 20% TPR had a positive effect on coffee purchase volume when compared to the effects of information and moral appeal.
Arce-Urriza, et al. (2017) [65]	Price	Supermarket/ grocery store Online retailer	Spain	6 mo (15 May 2007–15 November 2007)	Scanner/panel data	To evaluate the differential effect of price promotions on brand choice when shopping at a grocery store’s online outlet vs. brick-and-mortar store.	Brand choice	- Price promotions had a positive effect on purchases made in-person but not on purchases made online. - Frequent customers were more responsive to price promotions than infrequent customers.
Awan, et al. (2015) [80]	Price Promotion	Not specified	Pakistan	Not specified	Customer surveys	To identify which factors affect consumers’ decisions to purchase Halal food.	Purchase attitude	- Customers were influenced by Halal marketing and branding practices (e.g., sales promotions and celebrity endorsements). - Customers were willing to spend considerable effort and money to purchase Halal food as a result of Halal marketing.
Aziz, et al. (2013) [101]	Promotion	Other (shopping mall)	Malaysia	Not specified	Customer surveys	To determine the relationships between factors, including Halal marketing, and intention to purchase Halal products.	Purchase attitude	- Halal marketing promotion was positively related to purchase intention.
Banks et al. (2016) [93]	Placement	Convenience store	UK	Not specified	Marketing data	To describe the impact of endcap placement and shelf-ready cases for cookies sales.	Purchase volume Spending Market share	- Marketing efforts led to an increase in shoppers’ basket size (two-fold increase), spending (£3 increase), and market size (increased to £3.8bn) for cookies.
Bogomolova et al. (2019) [50]	Price	Supermarket/ grocery store	Australia	3 years (2 February 2012–31 December 2014)	Interviews/focus groups Loyalty card data	To assess reasons for first-time and impulse purchases	Product trialing Impulse purchasing	- The most common factor that prompted first-time brand purchases and impulse purchases was an item being placed on price promotion or having a special offer.
Breugelmans and Campo (2016) [63]	Price	Supermarket/ grocery store Online retailer	UK	78 weeks (July 2006–December 2007)	Scanner/panel data	To examine the cross-channel effects of price promotions (online vs. offline) on category purchase decisions.	Purchase incidence Purchase volume	- Price promotions had positive effects on purchasing decisions and degree of impact varied based on customer brand loyalty. - Promotions in one channel decreased category purchases in the other channel during the promotion period (online price promotions had a stronger impact on offline purchase decisions than vice versa). - High promotion frequency had negative effects on future promotion effectiveness.
Čábelková et al. (2015) [78]	Price	Supermarket/ grocery store	Czech Republic	2 months (October 2013–November 2013)	Customer surveys	To determine which activities are associated with customer store loyalty and differential effects by customer socio-demographic characteristics.	Customer loyalty	- Customer loyalty is linked to low prices and discount sales. - 44% of respondents said prices were one of the factors that compel them to make all their purchases in only one supermarket chain. - Probability of ranking prices and sales promotions as important factors was higher among older respondents and respondents who spent more monthly at supermarkets.
Caruso et al. (2018) [83]	Placement	Supermarket/ grocery store	Australia, New Zealand	56 hours (December 2008 and December 2015)	Direct observation	To assess how foot traffic and visual reach of endcaps differ by location.	Foot traffic Visual attention	- Back-of-store endcaps had 24% more foot traffic and 30% more visual reach than front-of-store endcaps.
Caspi et al. (2017) [84]	Placement Promotion	Dollar store Convenience store Other (pharmacy)	US	5 months (July 2014–November 2014)	Customer surveys Direct observation	To examine whether customers who shop at small/non-traditional food stores with more health promotions make healthier purchases.	Healthy eating index-2010 (HEI) score of products purchased	- Controlling for individual characteristics and store type, HEI scores for purchases were higher in stores with greater shelf space for fruits and vegetables. - Healthy advertisements on the store exterior were associated with lower purchase HEI scores. - The presence of interior healthy advertisements were not associated with purchase HEI scores.
Farrag (2012) [79]	Price	Supermarket/ grocery store	Egypt	Not specified	Interviews/focus groups	To measure to what extent compliance with Shariah moderates the relationship between sales promotion methods (price discount, product sampling, buy one get one free, sweepstakes/ lucky draws, scratch and win offers) of convenient products and consumer behaviors (product trial, stockpiling, spending more).	Purchase attitude	- Price discounts and buy-one-get-one were associated with self-reported stockpiling and spending more. - Price discounts had the strongest impact on consumer behavior (compared to sweepstakes/ lucky draw, scratch-and-win, free samples). - The relationship between price discounts and consumer behavior was moderated by Shariah law because some practices (e.g., scratch-and-win and sweepstake draws) were not compliant with Shariah law.
Felgate et al. (2012) [68]	Price	Supermarket/ grocery store	UK	86 weeks (29 May 2006–21 January 2008)	Scanner/panel data	To assess how supermarket loyalty card data can be used to analyze the effect of price promotions on spending.	Spending by product subgroup	- Promotions accounted for 14% of the variance in sales of beef. - While overall impact of promotion on sales of beef was insignificant, there was variability by cut of meat, customer group, and price promotions.
Fornari et al. (2013) [60]	Price	Supermarket/ grocery store	Italy	2011	Scanner/panel data	To assess the impact of different retailing-mix levers on private label market share.	Purchase volume	- Findings suggest partial support for price promotion increasing market share. - A significant presence on shelves, in width (increase in the number of product categories) and depth (increase in the number of SKUs in each product category) increased sales, suggesting that assortment is more important than price promotion.
Goić et al. (2011) [75]	Price	Supermarket/ grocery store	US	Not specified	Not specified	To investigate the effects of cross-market promotions (e.g., grocery store purchases that lead to price discounts for gas) on purchase volume and sales price.	Purchase volume Sales price	- Offering cross-market discounts on gas for grocery purchases led to an increase in both price and quantity of groceries purchased.
Guan et al. (2018) [54]	Price	Supermarket/ grocery store	US	2 years (2003–2005)	Scanner/panel data	To compare the effects of individually-targeted coupons for less healthful and more healthful foods on consumer purchasing patterns.	Purchase volume	- Being exposed to coupons resulted in an increase in the rate of purchase as compared to those without coupons. - People responded more to targeted coupons than to untargeted coupons. - Targeted coupons significantly increased purchases of both healthy and less healthy items, with greater increases in the purchases of less healthy items.
Hong et al. (2016) [94]	Placement	Supermarket/ grocery store	UK	Not specified	Scanner/panel data Direct observation	To examine whether the assortment or placement of one category affects purchase incidence in a different category that shares a common display space (e.g., frozen meals and ice cream).	Purchase incidence	- Consumers were less likely to purchase from a category of a given assortment when it was presented with another category assortment of greater variety and this effect was driven by the display proximity.
Huang et al. (2012) [57]	Price	Supermarket/ grocery store	US	Not specified	Customer surveysDirect observation	To identify shopper trip-level and point-of-purchase-level drivers of unplanned consideration and purchase behavior.	Purchase incidenceImpulse purchases	- An impulse purchase was more likely if a shopper viewed fewer product shelf displays, stood closer to the shelf, and referenced external information.
Jamal et al. (2012) [81]	Price	Supermarket/ grocery store	UK	Not specified	Interviews/focus groups	To investigate “ethnic” consumers’ responses to different sales promotions.	Perceived importance for purchase decisions	- “Ethnic” customers reported a range of responses to sales promotion—some were responsive, some hostile—depending on the “net worth” of the sales promotion.
Johnson et al. (2013) [58]	PlacementPrice	Supermarket/ grocery store	US	Not specified	Scanner/panel data	To examine how customized temporal discounts influence consumers’ decisions to purchase products and overall profit of the retailers.	Purchase incidence Brand choice Profit	- The customization of discounts by time and value yielded an increase in profits of 18–40% relative to a model that optimizes the value of the discounts.
Kacen et al. (2012) [62]	Placement Price	Supermarket/ grocery store	US	Not specified	Customer surveys	To assess the effect of retailing factors on the likelihood that a consumer will make an impulse purchase.	Impulse purchasing	- Products on sale and on display in a high–low pricing store increased the probability of an impulse buy to 7%. - A product had a 13.3% likelihood of being purchased if it was not on sale but a 17.6% likelihood if it was on sale. - A product had a 13.3% likelihood of being purchased if it was not on display, but a 20% likelihood if it was on display.
Kim et al. (2011) [66]	Price	Not specified	Japan	32 years (1976–2008)	Scanner/panel data Marketing data	To understand how changes among manufacturers in budget allocation from advertising to sales promotion affects sales volume and profitability.	Purchase volume profit	- Expenditure on sales promotion was associated with an increase in total volume sales but a decrease in profitability.
Leeflang et al. (2012) [70]	Price	Supermarket/ grocery store	Spain	1 year	Scanner/panel data	To determine the impact of price promotions in one category on the revenues of other categories.	Purchase volume Sales revenue	- Half of all price promotions expanded revenues for that category, especially for categories with deeper supported discounts. - There was a 61% probability that a price promotion affected sales of at least one other category. - Cross-promotional effects between categories more closely located in a store existed.
Levy and Gendel-Guterman (2012) [98]	Promotion	Supermarket/ grocery store	Not specified	Not specified	Customer surveys	To understand how consumer characteristics are correlated with advertising and the tendency to impulse buy store brands.	Impulse purchasing	- Advertising was positively correlated to the tendency to engage in impulse buying.
Liang et al. (2017) [82]	PromotionPrice	Other (organic market)	Taiwan	2 month (2012)	Customer surveys	To understand organic food consumers’ preferences for specific promotional programs (e.g., discounts, giveaways, limited time offers).	Purchase attitude	- Consumers preferred the programs in the discount category and the free giveaway category. - Limited time offers reduced purchase intention.
Mamiya et al. (2018) [71]	Price	Supermarket/ grocery store Convenience storeOther (pharmacy)	Canada	6 years (January 2008–December 2013)	Scanner/panel data	To assess whether there was a differential impact of price discounting of soda on sales by store-neighborhood education.	Purchase volume	- Across all levels of education and types of store, discounting was positively associated with soda sales. - The modification of the effect of price discounting by education was most prominent in pharmacies, where the average log sales associated with discounting increased as education decreased.
Minnema et al. (2017) [100]	Promotion	Supermarket/ grocery store	Netherlands	20 weeks (2010)	Scanner/panel data	To examine the effectiveness of instant reward programs with bonus premiums (i.e., collectible giveaways).	Shopping tripsCategory purchase incidence Brand choice Purchase volume	- Instant giveaway of collectible premiums resulted in increased brand and category choice probability, but no change in purchase quantity. - Consumers were more likely to choose the promoted brand if it was promoted with both the bonus premium and price discount compared to when it was promoted with just a price discount.
Mortimer and Weeks (2011) [77]	Price	Supermarket/ grocery store	Australia	Not specified	Customer surveys	To examine how price information is differentially considered by men and women in an Australian grocery store and how this affects grocery shopping behavior.	Purchase attitude	- The mean score for how consumers rate the importance of promotional pricing on their shopping decisions was 4.41 out of 5. - Men considered price attributes of products and promotional tactics as being significantly lower in importance than did women.
Mussol et al. (2019) [53]	Promotion Price	Supermarket/ grocery store	France	Not specified	Customer surveys	To explore in-store sales promotions as a tool in developing in-store relationships with consumers.	Purchase attitude	- Samplings, in-store games, lotteries nurtured consumer loyalty and relationships with brands. - Price-based promotions should be used to trigger purchases, whereas non-monetary promotions should be used to nurture brand relationships.
Nakamura et al. (2014) [85]	Placement	Supermarket/ grocery store	UK	Not specified	Scanner/panel data	To estimate of the effect of end-of-aisle display on sales.	Purchase volume	- End-of-aisle display increased sales volumes by 23.2% for beer, 33.6% for wine, and 46.1% for spirits, by 51.7% for carbonated drinks, 73.5% for coffee, and 113.8% for tea. - The effect size was equivalent to a decrease in price of between 4% and 9% per volume for alcohol categories, and a decrease in price of between 22% and 62% per volume for non-alcohol categories.
Nakamura et al. (2015) [6]	Price	Supermarket/ grocery store Convenience storeOther (various)	UK	1 years (2010)	Scanner/panel data	To investigate if consumers are more responsive to promotions on less-healthy products; and if there are socioeconomic differences in food purchases in response to price promotions.	Purchase volume	- After controlling for the reference price, price discount rate, and brand-specific effects, the sales uplift arising from price promotions was larger in less-healthy than in healthier categories. - A 1% increase in the depth of price discount led to a sales uplift by 1.44% within a given category.
Nordfält and Lange (2013) [99]	Promotion	Supermarket/ grocery store	Sweden	2 weeks (April 2008 and March 2009)	Scanner/Panel data Customer surveys	To investigate when and how in-store demonstrations work best.	Purchase volume	- In-store demonstrations increased sales, particularly when: closer to the weekend, the product was displayed next to the demonstration (235.07% increase), there was personnel offering the demonstration (24.31% increase), there was signage promoting the product (90.76% increase), and a commercial was run on an in-store TV (36.32%). - There was no significant change when in-store demonstrations were offered in a higher traffic area.
Osuna et al. (2016) [55]	Price	Supermarket/ grocery store	Not specified	2 years (2008–2009)	Loyalty card data	To explore how targeted coupons influence the uptake of new category and brand purchases.	Coupon redemption Product trialing	- To entice customers to buy in new categories, coupon redemption rates were higher for leading brands and categories that are popular, easy to store, have a low number of SKUs, and are frequently on sale. - To increase incremental purchases, coupons should be in categories that have low purchase frequency and high number of SKUs.
Page et al. (2019) [86]	Placement	Supermarket/ grocery store	Australia	24 hours	Direct observation	To explore the shopper traffic entering and exiting the middle aisle, and interaction with endcap promotions.	Shopper traffic Endcap use Basket size	- Overall use of endcaps in the store with a middle aisle was lower than that in the store with standard layout. - In a standard store, 2.2% of all observed shoppers were interacting with an endcap (48% at rear, 52% at front of store), while in the store with the middle aisle, 1.6%, (24% at the rear, 38% at the front, and 39% in the middle).
Panzone and Tiffin (2012) [61]	Price	Supermarket/ grocery store Convenience storeOther (liquor store)	England	Not specified	Customer surveys Receipts	To assess the impact of price promotions on wine on consumer purchases	Purchase volumePurchase initiation	- The presence of a discount was influential in determining what to buy (74% of the total impact of the discount), with a smaller effect on how much of a wine to buy (26% of the total impact), and no influence on interpurchase time. - Consumers primarily used discounts to determine the segment they will purchase from, and secondarily to purchase multiple units of the wine they had chosen.
Phillips et al. (2015) [87]	Placement Promotion	Supermarket/ grocery store	US	3 days	Direct observation	To explore whether the effectiveness of an end-of-aisle display is weakened if there is a product demonstration occurring near the end-of-aisle.	Visual attention	- The presence of an in-store demonstration near the end-of-aisle affected shoppers’ attention paid to the end-of-the-aisle. - The best way to attract attention to the end-of-aisle was not to have an in-store demonstration near it.
Phipps et al. (2010) [67]	Price	Supermarket/ grocery store	US	Not specified	Scanner/panel data Interview/focus groups	To explore the associations of discounted prices on supermarket purchases of selected high-calorie foods and more healthful, low-calorie foods.	Purchase volume Purchase attitude	- Odds of purchasing on price promotion compared with off promotion was 2.4 for high-calorie products and 1.2 for low-calorie products. - Odds of purchasing on sale versus full price were higher for sweet snacks, grain-based snacks, and sugar-sweetened beverages. - Participants emphasized the lure of sale items and said they took advantage of sales to stock up.
Point of Purchase Advertising International (2012) [15]	Placement	Supermarket/ grocery store	US	Not specified	Customer surveys Direct observation Other (store audit)	To investigate how shoppers are interacting with the in-store environment.	Purchase volume	- More than 1 in 6 purchases were made when a display with that brand was present in store.
Pozzi (2013) [64]	Price	Supermarket/ grocery store Online retailer	US	2 years (June 2004–June 2006)	Scanner/panel data	To assess if the introduction of e-commerce affects bulk purchase and stockpiling behavior by customers.	Purchase volume Impulse purchasing	- The share of expenditure on discounted items rose by 9–20% with the introduction of e-commerce. - Online shopping did not increase the likelihood of buying promoted items but positively impacted the amount customers bought when they bought promoted items. - The amount of purchasing increases as the amount of discount increases.
Ranjan (2018) [88]	Placement	Supermarket/ grocery store	US	8 months (1 May 2015–31 December 2015)	Scanner/panel data Loyalty card data Marketing data	To explore how category location, adjacencies, size and merchandizing determine consumers’ category choices.	Spending Purchase volume	- Moving to a central (peripheral) position in the layout improved purchase quantity and purchase incidence. - There was an overall “attention-stealing” effect of having neighbors.
Revoredo-Giha (2015) [69]	Price	Supermarket/ grocery store Convenience store Other (various)	UK	2006–2013	Scanner/panel data	To analyze the overall effect of price promotions on consumers’ food purchases.	Spending	- Price promotions had a positive effect on total household expenditure and expenditure by category across socioeconomic quintiles. - Consumers responded positively to price promotions on fruits, vegetables, soft drinks, juices, fats, and eggs.
Ruff et al. (2016) [89]	Placement Promotion	Convenience store	US	Not specified	Customer surveys Other (bag check)	To study how placement of products and signs in small convenience stores influence shopping behavior.	Purchase incidence	- Placement of water at eye-level and of produce in at the front of the store was not associated with sugar-sweetened beverage purchases. - Signs advertising water and sugar-sweetened beverages were not associated with sugar-sweetened beverage purchases.
Sanchez-Flack et al. (2017) [90]	Placement Promotion	Convenience store	US	1 years (2010)	Customer surveys Other (store audit)	To examine how product availability, placement, and promotion were associated with fruit and vegetable purchasing among Hispanic customers in San Diego County.	Purchase volume Spending	- Each additional square foot of display space dedicated to fruits and vegetables and each additional fresh fruits and vegetables display were associated with a $0.02 increase and $0.29 decrease, respectively, in fruit and vegetable purchasing.
Sano and Suzuki (2013) [72]	Price	Supermarket/ grocery store	Japan	1 months (May 2009–June 2009)	Scanner/panel data Other (shopping path)	To determine the share of product categories that should be included on discount flyers.	Purchase volume	- Price promotion of items would likely increase sales, particularly in some categories like drinks and western deli. - Price promotion would be less effective where there are already a lot of discounts.
Seva et al. (2011) [91]	Placement	Supermarket/ grocery store	Philippines	Not specified	Customer surveysDirect observation	To assess the effect of shelf position and product characteristics on the number and duration of eye fixations on a grocery shelf containing junk foods.	Visual attention	- Products placed at the top shelf received the highest attention from consumers as compared to the products placed on the other levels (the eye-level of majority of the subjects was in line with the top shelf). - Consumer attention decreased as the products’ vertical position deviated from eye-level.
Singh (2013) [73]	PromotionPrice	Supermarket/ grocery store	US	Not specified	Scanner/panel data	To investigate how pricing and promotion in frequently purchased categories influenced consumer visits to competing multiproduct grocery stores.	Store choice	- Own-store and cross-store prices, and own-store and cross-store feature advertising in frequently purchased categories impacted consumers’ choice. - For stockpiling categories, the own store feature activity (but not own store price) positively influenced consumer choice.
Smithson et al. (2015) [8]	Price	Supermarket/ grocery store Convenience store Other (various)	UK	52 weeks (December 2004–December 2005)	Scanner/panel data	To explore the role that price promotions play in purchasing levels of high-sugar food and drinks.	Purchase volume Brand switching	- 1/5 of foods and beverages bought on price promotion were purchased in addition to what would be expected for a given category if the price promotion was not in place. - Price promotions led to short-term brand switching. - Price promotions led to an overall increase in take-home food and drink volumes.
Spanjaard (2014) [95]	Promotion	Supermarket/ grocery store	Australia	Not specified	Customer surveys Direct observation Interviews/focus groups Other (diaries)	To understand which factors drive customer purchasing decisions.	Purchase attitude	- 41% of survey participants said they were influenced by promotional offers. - Trust and emotional connection the brand that are main purchasing decision drivers for customers.
Tacka (2019) [97]	Promotion	Not specified	US	5 days (19 September 2018–24 September 2018)	Customer surveys	To investigate the relationship between marketing activities (among other factors) and purchases of instant consumable snack foods	Purchase attitude	- Marketing activities were rated, on average, as being of “little importance” or “neither important nor unimportant,” when purchasing an instant consumable snack food.
Talukdar and Lindsey (2013) [74]	Price	Supermarket/ grocery store	US	52 weeks (2003–2004)	Scanner/panel data Customer surveys	To predict the effects of price changes on consumers’ food consumption behavior.	Purchase volume	- For healthy food, demand sensitivity was greater for a price increase than for a price decrease. - For unhealthy food, demand sensitivity was greater for a price decrease than a price increase.
Tan et al. (2018) [46]	Placement	Supermarket/ grocery store	Australia	Not specified	Scanner/panel data Direct observation	To compare the sales effectiveness of front versus back located end-of-aisle promotional displays in a supermarket.	Purchase volume	- Rear endcaps generated 416% sales uplift while front endcaps generated 346% sales uplift.
Tran (2019) [76]	PromotionPrice	Supermarket/ grocery store	Vietnam	2 weeks	Customer surveys	To investigate factors that influence customers’ impulse purchasing behavior.	Purchase attitude	- Sale promotion, presence of family and friends, emotion, merchandise display, money available and festival season accounted for 65.162% of impulse buying behavior.
Walmsley et al. (2018) [92]	Placement	Supermarket/ grocery store	England	170 weeks (January 2012–July 2017)	Scanner/panel data	To examine the effect of the store re-arrangements on purchasing of fruits and vegetables.	Purchase volume Spending	- The effect of the shop re-arrangement to make fruit and vegetables more prominent and moving the fruit and vegetable display to face the entrance led to an increase in sales and total dollars spent on fruits and vegetables.
Yildirim and Aydin (2012) [96]	Promotion	Supermarket/ grocery store	Turkey	10 days	Customer surveys	To assess the effect of supermarket announcements on customer behavior while shopping.	Purchase attitude	- Announcements related to price discounts, buy-one-get-one offers, membership deals, giveaways, and coupons were most desired and impactful announcements. - The most noticed type of announcement focused on price discounts.
Zhang (2017) [56]	Price	Online retailer	US	2 weeks (13 January 2014–26 January 2014)	Scanner/panel data	To evaluate the impact of coupons and informational nudges to customers identified through modeling on purchasing.	Purchase incidence	- Providing information and discounts to specific customers who are selected through modeling led to a higher conversion to purchase products.

## Appendix A

### Food and Beverage Retail Formats *:

• **Supermarkets:** A food retailer with greater than 9000 square feet of selling space and at least $2 million in annual sales. Non-food sales must account for 15% or less of total store sales (e.g., Kroger, Safeway).

• **Drug stores:** Drug stores feature prescription-based pharmacies but generate at least 20% of their total sales from other categories, including general merchandise and food (e.g., Rite-Aid, CVS).

• **Mass merchandisers:** Large department stores that sell primarily general merchandise and nonperishables but also carry a limited assortment of grocery products (e.g., K-Mart, Target).

• **Supercenters:** Also known as hypermarkets and superstores, supercenters are hybrid stores that combine mass merchandisers with full supermarkets (e.g., Walmart Supercenter, Fred Meyer).

• **Convenience and corner stores:** Convenience stores typically sell gasoline, general merchandise, alcohol and tobacco, and a limited selection of staple and ready-to-eat, prepared foods (e.g., 7-Eleven, Exxon).

• **Dollar stores:** Dollar stores typically emphasize low prices (many products cost $1) and offer little in the way of customer service. Traditionally, they focused on staple consumer goods and other nonfood items but, have increasingly offered food (e.g., Dollar Tree, Dollar General).

• **Club stores:** Also referred to as warehouse or volume stores, these are large-format outlets that specialize in selling food and selected general merchandise in bulk for relatively low prices, per unit. Consumers need paid memberships to shop at them (e.g., Costco, Sam’s Club).

**• Online retailers:** Retailers with an ecommerce presence, providing grocery click-and-collect or delivery services. These retailers can take any brick-and-mortar format (i.e., convenience stores, supermarkets, and mass merchandisers can all be online retailers).

• **Other:** Includes military commissaries, hospitals, and other food service providers, as well as direct-to-consumer food outlets such as farmers markets and community-supported agriculture.

* Definitions adapted from Volpe R, Kuhns A, and Jaenicke T. 2017. Store Formats and Patterns in Household Grocery Purchases, EIB-167, U.S. Department of Agriculture, Economic Research Service.

## Appendix B

### Search Terms

#### Research Question 1 Search String

((“Food” OR “Beverage”)

AND (“Manufacturer *” OR “Distributor *” OR “Supplier *” OR “Processor *” OR “grower *” OR “trade association *” OR “producer *” OR “industry” OR “company”)

AND (“Category captain *” OR “Category management” OR “Cooperative advertising” OR “Cooperative marketing” OR “dealer aid *” OR “fee *” OR “In-kind remuneration” OR “Pay-to-stay” OR “pouring right *” OR “Promotional allowance *” OR “Slotting” OR “Trade promotion” OR “Trade spend”)

AND (“Grocer *” OR “Grocery store *” OR “Supermarket *” OR “Food store *” OR “Online grocer * ” OR “Superstore *” OR “Warehouse *” OR “Club store *” OR “Convenience store *” OR “Food retailer *” OR “Food outlet *” OR “pharmac *” OR “corner store *” OR “discount store *” OR “dollar store *”)

NOT “pharmaceutical *”

#### Research Question 2 Search String

((“Food” OR “Beverage”)

AND (“Manufacturer *” OR “Distributor *” OR “Supplier *” OR “Processor *” OR “grower *” OR “trade association *” OR “producer *” OR “industry” OR “company”) OR (“Grocer *” OR “Grocery store *” OR “Supermarket *” OR “Food store *” OR “Online grocer * ” OR “Superstore *” OR “Warehouse *” OR “Club store *” OR “Convenience store *” OR “Food retailer *” OR “Food outlet *” OR “pharmac *” OR “corner store *” OR “discount store *” OR “dollar store *”))

AND (“Category captain *” OR “Category management” OR “Cooperative advertising” OR “Cooperative marketing” OR “dealer aid *” OR “fee *” OR “In-kind remuneration” OR “Pay-to-stay” OR “pouring right *” OR “Promotional allowance *” OR “Slotting” OR “Trade promotion” OR “Trade spend” OR “Buy-one-get-one” OR “buy one get one” OR “Circular *” OR “Coupon *” OR “Cross Promotion *” OR “Discount *” OR “Display *” OR “endcap” OR “Feature and display” OR “Incentive *” OR “In-store sampl” OR “loss leader *” OR “Placement” OR “POINT-of-sale advertis *” OR “Price Promotion *” OR “Rollback *” OR “Sales Promotion *” OR “Sign *” OR “Shipper *” OR “Shelf talker *” OR “Temporary price reduction *” OR “Two-for-one” OR “two for one”)

AND (“Customer” OR “Shopper” OR “Consumer”)

AND (“Behavior” OR “Preference” OR “Demand” OR “Consumption” OR “Choice” OR “decision” OR “purchas *” OR “attitude *” OR “willingness to pay”)

NOT (“pharmaceutical *”)

## Appendix C

**Table A1:** Newcastle–Ottawa quality scale adapted grading criteria.

Selection (max of 5 stars)	(1) Representativeness of the sample:	(a) Truly representative of the average in the target population^1^ (e.g., all subjects or random sampling)	*
		(b) Somewhat representative of the average in the target population^1^ (e.g., nonrandom sampling)	*
		(c) Selected group of users	No stars
		(d) No description of the sampling strategy	No stars
	(2) Sample size:	(a) Justified and satisfactory	*
		(b) Not justified	No stars
	(3) Non-respondents:	(a) Comparability between respondents’ and non-respondents’ characteristics is established, and the response rate is satisfactory	*
		(b) The response rate is unsatisfactory, or the comparability between respondents and non-respondents is unsatisfactory	No stars
		(c) No description of the response rate or the characteristics of the responders and the non-responders	No stars
		(d) Not applicable (e.g., aggregate sales data)	NA
	(4) Ascertainment of risk factor:	(a) Built into dataset	**
		(b) Built into study design	**
		(c) Self-reported/-stated	*
		(d) No information disclosed	No stars
Comparability (max of 2 stars)	(1) The subjects in different outcome groups are comparable, based on the study design or analysis. Confounding factors are controlled	(a) The study controls for the most important factor (e.g., income/SES)	*
		(b) The study controls for any additional factor (e.g., age, gender, household size, race)	*
		(c) Not applicable (e.g., there is no comparison group)	NA
Outcome (max of 3 stars)	(1) Assessment of the outcome:	(a) Independent blind assessment	**
		(b) Record linkage	**
		(c) Self reports	*
		(d) No description	No stars
	(2) Statistical test:	(a) The statistical test used to analyze the data is clearly described and appropriate, and the measurement of the association is presented, including confidence intervals and the probability level (p value)	*
		(b) The statistical test is not appropriate, not described or incomplete	No stars

“Target population” defined based on authors’ definition of their “target population.” The Newcastle–Ottawa quality scale assigns studies composite quality scores by awarding up to nine stars. A study can be awarded a maximum of one star (*) in the categories of: representativeness of the sample, sample size, non-respondents, and statistical test. A maximum of two stars (**) can be awarded in the categories of: ascertainment of risk factor, comparability, and assessment of outcome.

## Appendix D

**Table A2:** Quality assessment of the included studies using the Newcastle–Ottawa quality scale (*n* = 54).

Reference	Selection				Comparability	Outcome		
	Representativeness of the Sample	Sample Size	Non- Respondents	Ascertainment of Risk Factor	Are Confounding Factors Controlled	Assessment of Outcome	Statistical Test	Overall Score *
Andorfer et al. (2015)	0	1	0	2	1	1	1	6/10
Arce-Urriza et al. (2017)	1	1	NA	2	NA	2	1	7/7
Awan et al. (2015)	1	0	0	1	NA	1	1	4/8
Aziz et al. (2013)	0	0	0	1	0	1	1	3/10*
Banks et al. (2016)	0	0	NA	1	NA	1	0	2/7*
Bogomolova et al. (2019)	1	1	NA	1	2	1	1	7/9
Breugelmans and Campo (2016)	1	1	NA	2	1	2	1	8/9
Čábelková et al. (2015)	1	1	0	1	2	1	1	7/10
Huang et al. (2012)	1	0	0	2	2	2	1	8/10
Caruso et al. (2018)	1	1	0	2	0	2	1	7/10
Caspi et al. (2017)	1	0	0	1	1	1	1	5/10
Farrag (2012)	0	0	0	2	0	1	1	4/10
Felgate et al. (2012)	1	1	NA	2	0	2	1	7/9
Fornari et al. (2013)	1	0	NA	2	NA	2	1	6/7
Goić et al. (2011)	0	0	NA	2	NA	0	1	3/7
Guan et al. (2018)	1	1	0	2	2	1	1	8/10
Hong et al. (2016)	1	1	NA	2	1	2	1	8/9
Jamal et al. (2012)	1	1	0	1	NA	1	0	4/8
Johnson et al. (2013)	1	0	0	2	NA	2	1	6/8
Kacen et al. (2012)	1	0	NA	2	2	1	1	7/9
Kim et al. (2011)	1	1	NA	2	NA	2	1	7/7
Leeflang et al. (2012)	0	0	NA	2	NA	2	1	5/7
Levy and Gendel-Guterman (2012)	1	1	0	1	2	1	1	7/10
Liang et al. (2017)	1	1	NA	2	0	2	1	7/9
Mamiya et al. (2018)	1	1	NA	2	2	2	1	9/9
Minnema et al. (2017)	1	1	0	2	2	2	1	9/10
Mortimer and Weeks (2011)	1	1	0	2	1	1	1	7/10
Mussol et al. (2019)	1	0	0	2	1	1	1	6/10
Nakamura et al. (2015)	1	1	0	2	1	2	1	8/10
Nakamura et al. (2014)	1	0	NA	2	NA	2	1	6/7
Nordfält and Lange (2013)	1	0	NA	2	NA	2	1	6/7
Osuna et al. (2016)	1	0	NA	2	1	2	1	7/9
Page et al. (2019)	1	0	NA	2	2	2	1	8/9
Panzone and Tiffin (2012)	1	1	0	1	0	1	1	5/10
Phillips et al. (2015)	1	0	NA	2	1	2	1	7/9
Phipps et al (2010)	0	0	0	2	1	2	1	6/10
Point of Purchase Advertising International (2012)	1	1	0	2	0	2	0	6/10
Pozzi (2013)	1	1	NA	2	2	2	1	9/9
Ranjan (2018)	1	0	NA	2	1	2	1	7/9
Revoredo-Giha (2015)	1	1	0	2	1	2	1	8/10
Ruff et al. (2016)	1	0	0	2	2	1	1	7/10
Sanchez-Flack et al. (2017)	1	1	1	2	2	1	1	9/10
Sano and Suzuki (2013)	0	0	NA	2	NA	2	1	5/9
Seva et al. (2011)	0	0	0	2	0	2	1	5/10
Singh (2013)	1	0	0	2	2	2	1	8/10
Smithson et al. (2015)	1	1	0	2	2	2	1	9/10
Spanjaard (2014)	1	1	0	1	NA	1	NA	4/7
Tacka (2019)	1	0	0	1	0	1	1	4/10
Talukdar and Lindsey (2013)	1	0	NA	2	2	2	1	8/9
Tan et al. (2018)	1	0	NA	2	1	2	1	7/9
Tran (2019)	0	0	0	1	0	1	1	3/10*
Walmsley et al. (2018)	1	1	NA	2	0	2	1	7/9
Yildirim and Aydin (2012)	1	1	0	2	1	1	1	7/10
Zhang (2017)	1	1	NA	2	2	2	1	9/9

* Indicates low quality.
